# Vulnerability detection in Java source code using a quantum convolutional neural network with self-attentive pooling, deep sequence, and graph-based hybrid feature extraction

**DOI:** 10.1038/s41598-024-56871-z

**Published:** 2024-03-28

**Authors:** Shumaila Hussain, Muhammad Nadeem, Junaid Baber, Mohammed Hamdi, Adel Rajab, Mana Saleh Al Reshan, Asadullah Shaikh

**Affiliations:** 1 Department of Computer Science, Sardar Bahadur Khan Women’s University, Quetta, Pakistan; 2 Department of Computer Science and IT, University of Balochistan, Quetta, Pakistan; 3 Higher Colleges of Technology, Abu Dhabi, United Arab Emirates; 4 GIPSA-Lab, University Grenoble Alpes, 38000 Grenoble, France; 5 Department of Computer Science, College of Computer Science and Information Systems, Najran University, 61441 Najran, Saudi Arabia; 6 Department of Information Systems, College of Computer Science and Information Systems, Najran University, 61441 Najran, Saudi Arabia

**Keywords:** Vulnerability detection, Self-attentive QCNN, Feature extraction, Hybrid GCN, Software security, CodeBERT, Engineering, Mathematics and computing

## Abstract

Software vulnerabilities pose a significant threat to system security, necessitating effective automatic detection methods. Current techniques face challenges such as dependency issues, language bias, and coarse detection granularity. This study presents a novel deep learning-based vulnerability detection system for Java code. Leveraging hybrid feature extraction through graph and sequence-based techniques enhances semantic and syntactic understanding. The system utilizes control flow graphs (CFG), abstract syntax trees (AST), program dependencies (PD), and greedy longest-match first vectorization for graph representation. A hybrid neural network (GCN-RFEMLP) and the pre-trained CodeBERT model extract features, feeding them into a quantum convolutional neural network with self-attentive pooling. The system addresses issues like long-term information dependency and coarse detection granularity, employing intermediate code representation and inter-procedural slice code. To mitigate language bias, a benchmark software assurance reference dataset is employed. Evaluations demonstrate the system's superiority, achieving 99.2% accuracy in detecting vulnerabilities, outperforming benchmark methods. The proposed approach comprehensively addresses vulnerabilities, including improper input validation, missing authorizations, buffer overflow, cross-site scripting, and SQL injection attacks listed by common weakness enumeration (CWE).

## Introduction

The COVID-19 pandemic has dramatically intensified the use of computer applications, leading to an unprecedented increase in software vulnerabilities. According to the national vulnerability database (NVD), there were 20,158 reported vulnerabilities in 2021^1^. This exponential growth in security vulnerability is causing significant economic impacts and substantial financial losses^2–7^.

Therefore, software vulnerability detection has become more crucial and challenging than ever. The need for generalized, scalable, accurate, fine-grained, and high-speed automatic vulnerability detection approaches is evident. Vulnerability typically stems from programming oversights, which the current detection tools, using either static or dynamic code analysis, often fail to address adequately. The analysis of code for security vulnerability without execution is the static code analysis technique, while in dynamic code analysis, the running application is tested for security vulnerability. Static code analysis techniques can be resource-intensive, while dynamic code analysis can increase execution time and negatively impact performance^8^. Both of these approaches are language-specific, rule-based, and dependent on the knowledge of the developers, making them prone to errors, biased, coarse-grained, and leading to unacceptably high false-negative rates.

Machine learning (ML) techniques have proven promising in vulnerability assessment^9–14^. The deep neural networks (DNNs) have demonstrated capabilities in learning source code patterns, excelling in syntax-level bug detection and pattern recognition^15–17^. However, existing deep learning (DL) solutions for vulnerability assessments have certain limitations; they primarily concentrate on the syntactic structure of code, neglecting its semantic information^18–20^. They target either a single file of source code or a small dataset or rely on application processing interface APIs to address the selected vulnerability. Furthermore, DL techniques often struggle to understand the value transfers within source codes due to a lack of semantic information, resulting in a high false-positive rate and less scalable approach^21–23^.

The employed self-attentive quantum convolutional neural network along with deep learning techniques significantly improves the memory bottleneck issue, semantics understanding of code pattern and accelerated the performance. The proposed vulnerability detection system can detect a range of vulnerabilities, including improper input validation, SQL injection attacks, missing authorization, cross-site scripting, and buffer overflow attacks listed among the top 25 most impactful security vulnerabilities by common weaknesses enumeration (CWE). The CWE is a project of Mitre and is responsible for listing the software and hardware weakness types according to their impact to help prevent the vulnerability. This research paper contributes to the field of automatic vulnerability detection in several significant ways:

1. It develops a novel vulnerability detection system that implies efficient and accurate vulnerability detection using hybrid feature extraction by concatenating graph-based and sequence-based approaches coping with complex vulnerability patterns, enhancing vulnerability detection granularity, and reducing false-positive rates.
2. It proposes a hybrid graph neural network based on GCN-RFEMLP to overcome the absence of order information of nodes in the graph. Our fused wrapper method has reduced the dimension of features and removed irrelevant features to improve efficiency.
3. It introduces bimodal pre-trained CodeBERT model to implement fine-tuned feature extraction, reducing thereby, the semantic gap to improve vulnerability detection.
4. It analyzes the vulnerability detection dataset and balances the dataset to avoid overfitting, thereby improving the performance.
5. It employs the benchmark comprehensive software assurance reference dataset (SARD) for model training and testing, preprocessing the datasets to achieve optimized results. The proposed system is tested with five different datasets to ensure its performance, robustness, and validity.
6. It employs novel quantum convolutional neural network using self-attentive pooling to improve the computation, long-term dependencies, and memory bottleneck issues to classify the vulnerable code and type of vulnerability. To the best of our knowledge, QCNN-Self Attentive pooling is used for the first time to classify the vulnerabilities.
7. It proposes a novel framework for effective feature selection, contributing to a broader understanding of this field and suggesting a more balanced and effective approach to vulnerability detection across diverse types.

The remainder of this paper is structured as follows:

"Related work" section delves into a review of relevant literature. "Methodology" section outlines the methodology employed in this research. "Experiments and results" section details the experiment and results, including the experimental setup and derived results from the proposed method. "Conclusion" section offers the conclusions drawn from this study.

## Related work

Manual source code auditing, involving a team of security experts, scrutinizing source code for vulnerability, is the most traditional approach to finding software vulnerability^24^. However, conventional software vulnerability analysis techniques often struggle to cope with real-time and ever-increasing software security vulnerability.

Vulnerability detection based on code analysis is trending and is classified into three main approaches: static, hybrid, and dynamic vulnerability detection^25^. Static analysis scrutinizes source code without execution, whereas dynamic analysis examines it through execution. The hybrid analysis combines the two. Many tools and techniques, such as code comparison, symbolic execution, and inference techniques, have been developed for static analysis. However, these techniques do not cover all existing vulnerabilities and are ill-equipped to analyze emerging security threats. Dynamic analysis techniques, including fuzzing and taint analysis, require substantial computational time and resources^26–31^. Furthermore, the performance and reliability of these methods are insufficient to meet current security challenges.

**Figure 1:**
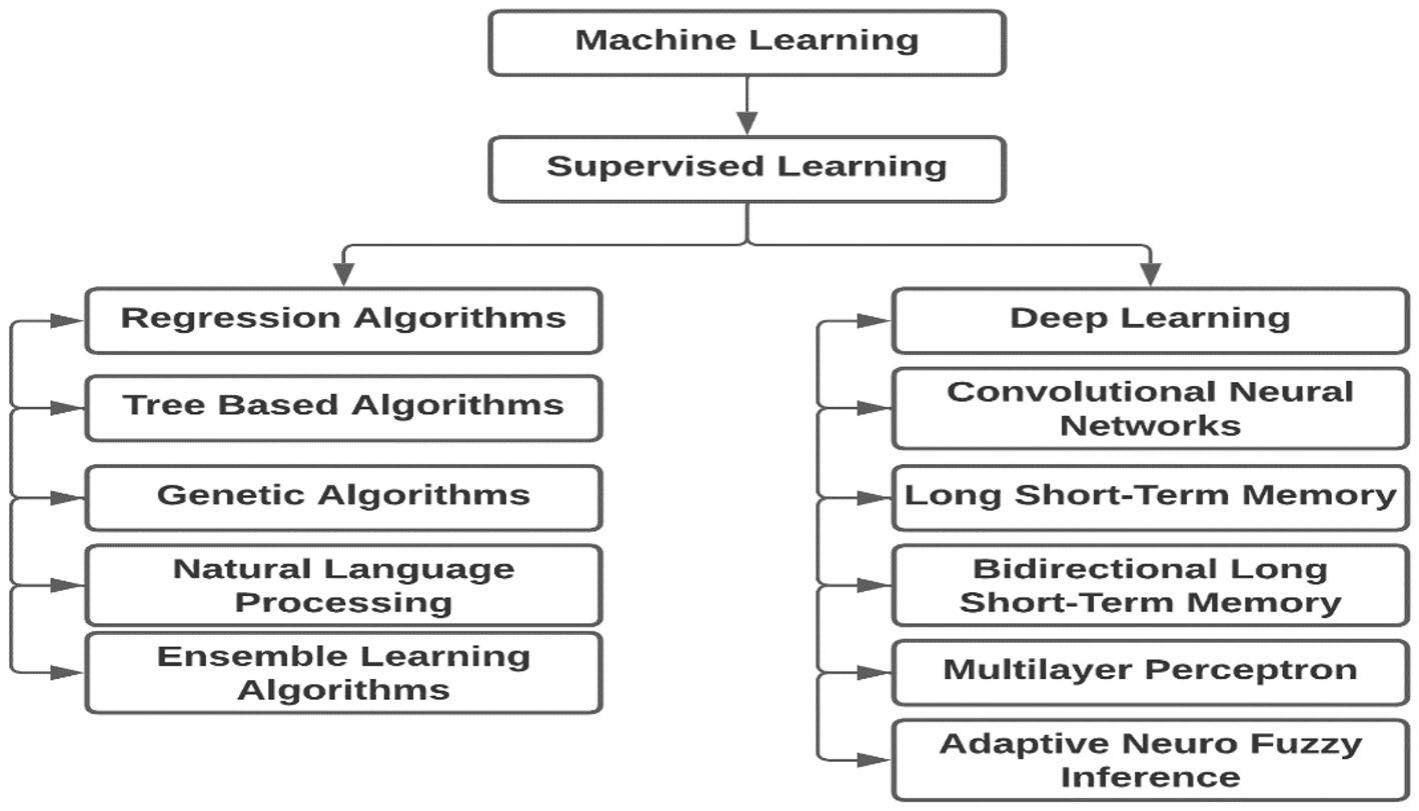
Machine learning techniques used for vulnerability assessments.

The surge in software vulnerability has driven researchers to devise better detection strategies. Software security researchers have begun leveraging machine learning's predictive power to address these security challenges. Machine learning techniques, whether supervised, unsupervised, or semi-supervised, are increasingly used for vulnerability detection. Among various machine learning approaches, supervised machine learning is widely adopted for software vulnerability detection. Figure 1 illustrates the supervised machine learning approaches for vulnerability assessment.

### Code representation learning

The code must follow a specific format to implement machine learning techniques, categorized into three primary representation methods:

*Sequence-based* In this approach, data is divided into chunks, such as characters, tokens, or APIs, utilizing techniques like bag-of-words, n-gram, word2vec, etc. These techniques involve data preprocessing, tokenization, and the adoption of neural networks. However, they may lack long-term contextual code abstraction.

*Tree-based* This method employs a neural network structure on abstract syntax tree (AST)-based data representation. The tree is subdivided into small statements containing code snippets. Challenges include code fragment complexity and gradient vanishing.

*Graph-based* This approach represents code in a graph structure, primarily using a code property graph (CPG) composed of an abstract syntax tree (AST), control flow graph (CFG), context flow graph (XFG), and program dependency graph (PDG) for intermediate code representation^32^. While graph-based techniques can address long-term dependency issues, they require intensive computation.

In one of the related researches, a vulnerability analysis study used graph neural networks (GNN) and circle-gated graph neural networks to detect the vulnerable code^33,34^. In another study, the researchers used a flow graph for source code representation, performed vectorization through word2vec, and applied the graph neural network method to identify the vulnerability^35–37^. The software vulnerability detector named DeepVulSeeker used a pre-trained model to convert natural language descriptions to programming code. Another research study in context used intermediate code representation by applying AST, CFG, and DFG and deployed a pre-trained model, while CNN and FNN neural networks were used to classify the vulnerability^38,39^. The abstract syntax tree neural networks^40,41^ and self-attentive deep neural network coupled with text mining were also tried^42^. Similarly, ChatGPT involves human interaction to identify vulnerabilities and recommend fixes^43^.

Another study explored regression trees for vulnerability detection^44^. Similarly, a hybrid approach using deep learning-based lightweight-assisted vulnerability was used in a study pertaining to the same, while another research used minimum intermediate representation learning^45,46^. The researchers exploit program slicing and binary gated recurrent unit (BGRU) in a similar nature of study, while code slicing using code metrics as features is used to detect vulnerabilities related to pointer usage^47,48^. Other studies implemented deep learning techniques like CNN and others, along with feature selection, for detecting SQL and cross-site scripting vulnerability^48–51^. Yet another study proposed a model based on source feature learning and classification^52^. It has been observed that feature selection is frequently studied alongside machine learning approaches for vulnerability detection^53–55^.

Two similar studies used word2vec and LSTM to identify code with cross-site scripting, SQL injection, cross-site forgery, and open redirect vulnerability^56,57^. The recurring neural network model called BiLSTM is used to focus on buffer errors and resource management vulnerability detection^58^. Similarly, BiLSTM and taint analysis performed well in one of the research pursuits conducted in the same context^59^. Techniques like CNN, long-short-term memory (LSTM), and directed graphs were used for vulnerability detection^60^.

One of the related studies in this regard compared the Random forest, CNN, and RNN techniques to benchmark vulnerability detection^61^. Similarly, the GNN-based model outperformed for vulnerability detection^62,63^. Another study presented a comparative analysis using Naïve Bayes, decision trees, SVM, k-nearest neighbor, and RF to evaluate software vulnerability detection performance^64–67^. Yet another study focusing on SVM, multinomial Naïve Bayes classifiers, and bidirectional encoders based on BERT transfer learning concluded that BERT outperformed other methods in detecting vulnerability^68^. Notably, none of the studies reviewed considered the semantic similarity of code, prominent the gap in the deep learning techniques used for vulnerability detection. In contrast, our work extracts the semantic similarity of the code, enhancing system performance, as further detailed in the results section.

Improper input validation, a major cause of security vulnerability in computing applications, can trigger SQL injection attacks, missing authorization, cross-site scripting (XSS) attacks, and buffer overflows. The Common Weakness Enumeration (CWE) project of the Mitre organization, a comprehensive dictionary of software weaknesses, ranked input validation as the fourth most frequently occurring and dangerous security vulnerability in 2021^69,70^. Therefore, we selected improper input validation, cross-site scripting, buffer overflow, missing authorization, and SQL injection vulnerability ranked among the top 25 most impactfull and dangerous security vulnerabilities listed by CWE for evaluating our proposed system. Table 1 below shows some vulnerability detection techniques commonly used to analyze the selected vulnerability.

**Table 1:** Commonly used techniques for vulnerability detection.

Vulnerability	Approach
Cross-site scripting XSS attacks	1. Cross-site scripting attack XSS detection using a modified CNN model^71^ 2. Automated server-side XSS attack detection using boundary injection^72^ 3. Taint tracking-based analysis of DOM cross-site scripting named as TT-XSS^73^ 4. Support Vector Machine is used to detect blind cross-site scripting vulnerability^74^ 5. Reducing attack surfaces for cross-site scripting attacks using secure SDLC^75^ 6. Detecting cross-site scripting vulnerability using LSTM and recurrent neural networks (RNN) named DeepXSS^76^ 7. Using genetic algorithms and reinforcement learning for XSS attack detection^77^ 8. Using ML with hybrid features for XSS attack detection^78^ 9. Using Fuzzy inference for dynamic detection of XSS cross-site scripting attacks^79^
Buffer overflow attacks	1. Analyzing network intrusion for buffer overflow attacks^80^ 2. Implementing string library function to detect integer overflow-to-buffer overflow attacks^81^ 3. Performed static buffer overflow detection and suggested automatic detection^82^ 4. Static buffer overflow detection and repair using the Bovlnspector tool^83^
SQL injection attacks	1. SQL injection attacks detection using a decision tree^84^ 2. Using behavior and response analysis for SQL injection attacks^85^ 3. SQL injection attack detection in web applications using heuristic-based analysis^86^ 4. Applying neuro-fuzzy techniques to prevent and detect SQL injection attacks^87^ 5. Algorithm designed for black box testing to mitigate SQL injection vulnerability^88^ 6. A traffic-based technique called DIAVA to detect data leakages and SQL injection attacks^89^ 7. A hybrid method consists of augmenting database tables with symbols, then using an algorithm for queries and another algorithm designed for string matching to prevent and detect SQL injection attacks^90^ 8. Using intrusion set randomization to detect SQL injection attacks^91^ 9. A tool is developed to detect SQL injection attacks and display suggestions to fix them^92^
Missing authorization	1. The tool is developed to detect missing authorization in distributed cloud systems using inferring variable definition, user-owned data, and critical system state^93^ 2. The proposed role cast SE-based technique consists of the context of security-sensitive events that are control-dependent on roles^94^ 3. The Vanguard is an approach consisting of static analysis for sensitive operations, analyzing sustainability using taint analysis, and the existence of risk degree of missing authorization^95^ 4. VRust is proposed to analyze vulnerability, including missing authorization for Solana, by assigning validation rules for vulnerable input accounts^96^ 5. The CRIX system consists of interprocedural, semantic, and context-aware systems^97^ 6. MACE is based on checking the authorization state consistency^98^

## Methodology

This section describes our proposed system for vulnerability detection, which introduces fused feature extraction that leverages semantic and syntax understanding of code for a nuanced vulnerability assessment.

### Framework of proposed vulnerability detection system

Code auditing is performed predominantly on C/C+ languages, while there is always a space for Java code auditing due to deficient code auditing techniques quantified for this language. Our system aims to automatically detect software vulnerability from Java code using DL, considering syntactic structure and code semantics, focusing on fine-grained vulnerability detection. Given that existing DL techniques often overlook the semantic relationships in code, our system is designed to fill this gap and improve the false-positive rate. The proposed system uses a novel mechanism based on hybrid feature extraction that concatenates sequence-based and graph-based feature extraction and detects the vulnerability using deep learning.

**Figure 2:**
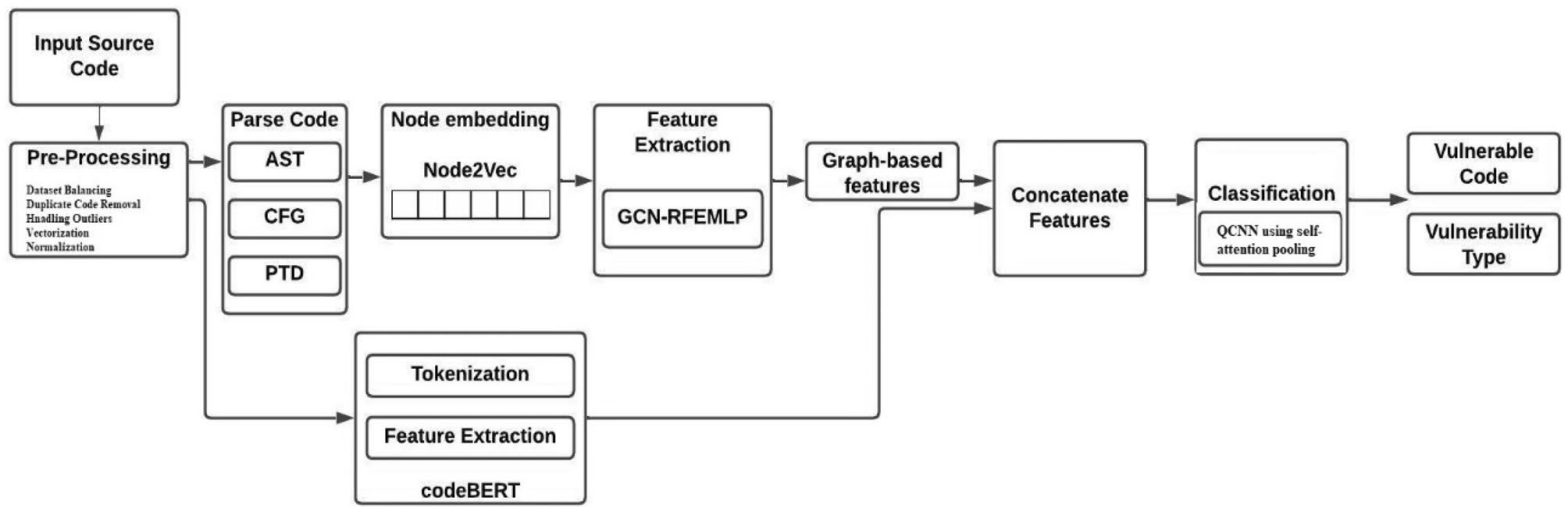
The framework of the proposed vulnerability detection system.

The proposed methodology is depicted in Fig. 2 given below. The proposed scheme is divided into three parts (1) Intermediate input representation (2) Hybrid feature extraction, and (3) Classification. The first step comprises a standard dataset converted into source code representation using code property graph and tokenization to get it presentable to leverage machine learning techniques. In the second step, the hybrid feature extraction is applied. The graph feature extraction used along with sequence-based feature extraction leverages the semantic and syntax structure of code. The extracted features are concatenated, and a quantum convolutional neural network with self-attentive pooling is employed to detect selected vulnerabilities.

The selected vulnerabilities are listed among the most impactful according to CWE and include improper input validation, SQL injection vulnerability, missing authorization, cross-site scripting, and buffer overflow. The system detects vulnerable functions and types of vulnerability.

### Dataset/data acquisition

To train our proposed system, we have used the Software Assurance Reference Dataset (SARD) benchmark dataset, which contains hundreds of thousands of source code programs with known vulnerabilities. This dataset includes 42,212 files comprising 29,258 safe samples and 12,954 unsafe samples of source code, covering 150 classes of bugs or weaknesses listed by CWE^99–104^. For our study, we have selected 46,447 Java programs from SARD, including vulnerabilities related to SQL injection attacks, missing authorization, cross-site scripting, improper input validation, and buffer overflow. The proposed system is validated using other benchmark datasets, including Juliet java 1.3^105–107^, FUNDED, Vul4j, CVEfixes, and CodeXGLUE.

### Dataset preprocessing

Data preprocessing involves several essential steps.

- Dataset balancing.
  Addressing dataset imbalance is crucial for the optimal performance of machine learning algorithms. The benchmark dataset for vulnerability detection often exhibits a significant disparity between vulnerable and clean codes. Achieving a balanced dataset is vital for accurate and efficient algorithm performance, helping reduce false positive ratios. Additionally, missing values are appropriately handled.
- Duplicate code removal
  Removing duplicate code enhances performance, reduces complexity, and minimizes execution time. Decision trees are employed for the efficient removal of duplicate code and code clones.
- Handling outliers
  Organizing the dataset is essential for improved performance. Outliers are detected and effectively handled using log transformation, contributing to dataset normalization.
- Vectorization
  Textual data is transformed into numerical form through vectorization, ensuring uniform scaling and enhancing algorithm performance.
- Normalizing
  Further normalization of the dataset ensures consistent scaling without compromising range differences. Data normalization equalizes the impact of each feature, addressing potential accuracy issues arising from inherently large values. The Z-Scaling technique is employed for data normalization, converting text-based datasets into integers.

### Graphical feature extraction

#### Intermediate code representation

We have applied the classical code property graph (CPG) for graphical code representation, which is a combination of abstract syntax tree (AST), control flow graph (CFG), and program dependency graph. It helps analyze the syntactic structure and code semantics. It is important to convert the code into intermediate code representation to remove the pointless points and reduce the dependencies.

- Abstract Syntax Tree (AST)
  The AST is used to parse the syntactic structure of code effectively. The abstract syntax tree comprises a root node that holds functions, branches of statements, declarations, predictions, and expressions while the leaf nodes represent the operators, identifiers, and keywords.
- Control Flow Graph (CFG)
  The CFG represents the order of code execution. It expounds statements and conditions that need to be met for the execution of code branches. The nodes in the CFG indicate the statements, while the edges denote the transfer of control.
- Program Dependency Graph (PDG)
  It describes the control and data dependencies in the function. The data dependency edge holds the declared variable to be used later, while the control dependency edges denote the impact of predicates on variables.

#### Node embedding

**Figure 3:**
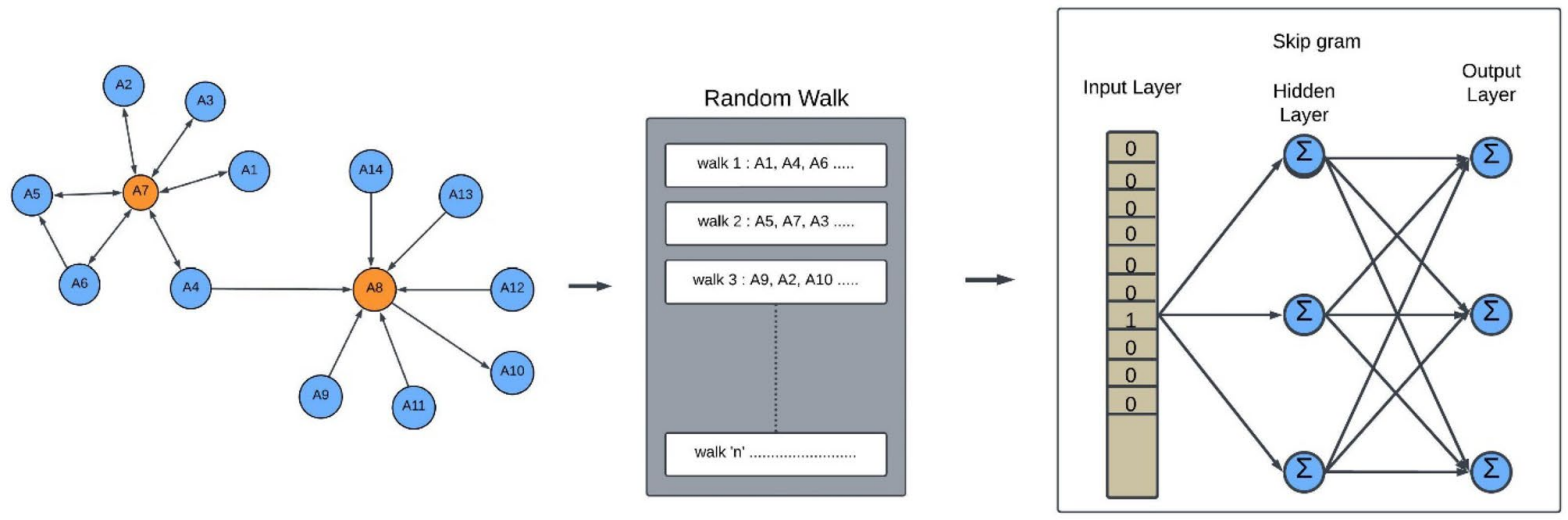
Structure of Node2Vec using random walk and skip-gram.

Node embedding aims to reduce the nodes' properties in smaller dimension vectors. The outcome of node embedding is fed as input to downstream machine learning-based processing techniques. Flexibility in exploring neighborhoods in node2vec has been observed to provide a richer representation. The rich structural information improves the ability of features to imply nonlinear information. Therefore, the node2vec is used for node embedding with random walk using skip-gram with negative sampling technique to maximize the probability of preserving the neighborhood of nodes. The node2vec is a second-order Markov chain. It implements random walk on graphs to extract the context pair using bootstrapping approach and use them for training the word2vec model. It transforms graphs to numerical representation while preserving the structure of the network in a way that the close nodes remain close in embedding. The structure of node2vec is given in Fig. 3.

#### Feature extraction

**Figure 4:**
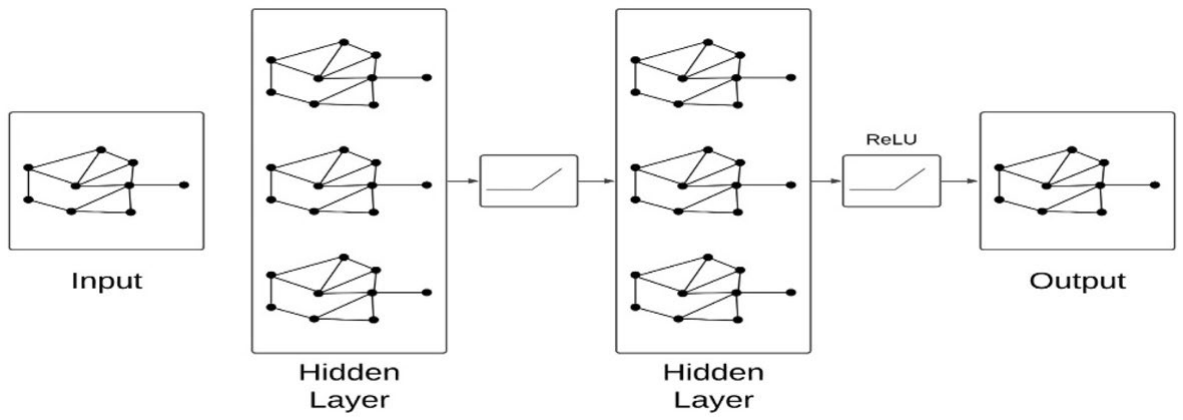
Structure of graph convolutional neural network.

We have employed hybrid graph neural network GCN-RFEMLP based on graph convolutional neural network (GCN) and multilayer perceptron fused with recursive feature elimination wrapper. The GCN lacks feature similarity, which can create noise. We, therefore, have concatenated RFEMLP with GCN to overcome this issue. The graph convolutional neural network is designed to deal with graph structure data. It implements a message-passing technique where the embedding information of a node is updated based on the neighboring node. The node embedding is converted into graph embedding, serving as input to a fully connected classifier. We have added a bi-affine layer in GCN to achieve better dependency parsing and preserve code semantics. The structural composition of graph convolutional neural networks is illustrated in Fig. 4 given below.

We used an MLP neural network with a rectified linear activation function, ReLu, on the hidden layer and a Softmax activation function on the output layer. The generalized formula for ReLu is depicted in Eq. (1).

1 $${\text{O }} = {\text{ WA}} + {\text{B}}$$

where O is the output before applying the activation function, W represents the weights, A represents the input to the layer, and B represents the bias.

**Figure 5:**
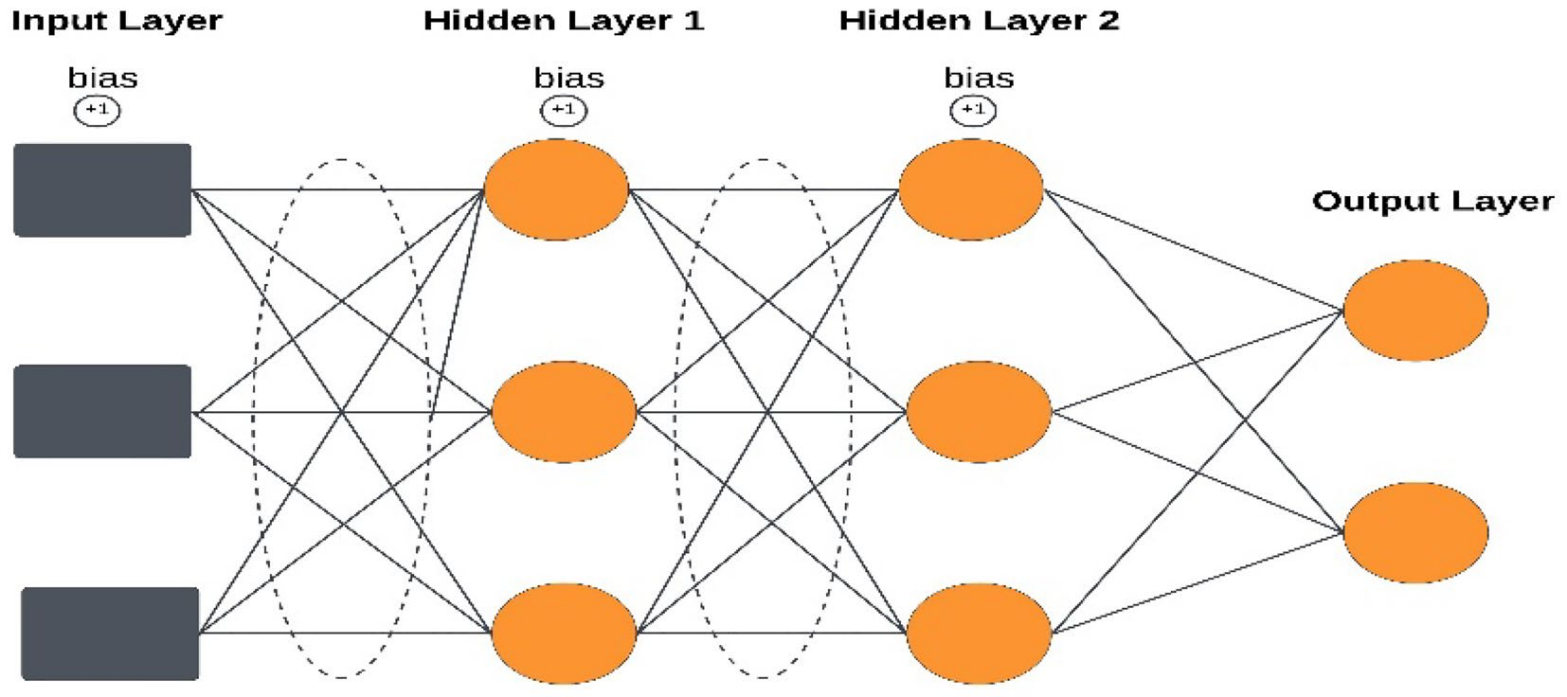
Structure of multilayer perceptron neural network model (MLP).

The Fig. 5 illustrates structural composition of MLP network. We have used adam, adadelta, momentum, and stochastic gradient descent (SDG) optimizer along with loss functions mean square error (MSE) and mean absolute error (MAE) to select the best fit. We have paired each optimizer with a loss function to get the results. The selections given below show the combination of each optimizer and loss function. Selection 1 shows the combination of the adam optimizer with the MSE loss function similarly; Selection 2 shows the combination of the adam optimizer with the MAE loss function, and so on.

Table 2 depicts different compositions of optimizers and loss functions. The results obtained from each selection are compared to implement the best combination of optimizer and loss function to improve the system's accuracy. We have conducted experiments to acquire the optimal combination with minimal loss to improve the algorithm's performance. The loss function enumerates the difference between the actual value and the predicted value. The selection 3 and selection 7 showed improved results. We, therefore, have selected selection 7 to use with MLP to boost the performance. Moreover, the model training contains regulating the parameters, hyper-parameter tuning, CommitCount functions, setting bias, optimizers, loss functions, and weights to reduce false positive rate. The fine-tuned model detects the vulnerability. The specified learning rate set in the proposed model is 0.0005 on 300 epochs, neurons = 128, early stopping = 30, and batch size = 64. The RFEMLP imposes a machine learning-based wrapper technique called recursive feature elimination (RFE) on a multilayer perceptron neural network. The RFE keeps on eliminating the irrelevant feature on each iteration until it reaches the most impactful features. The RFE reduces the redundant features to improve efficiency. We have implemented a decision tree classifier for RFE. Based on the aggregate difference between the features space, we have set the ranking of features from the most important to the least important.

**Table 2:** Different combinations of optimizers and loss functions.

No	Combination
Selection 1	Adam + MSE
Selection 2	Adam + MAE
Selection 3	AdaDelta + MSE
Selection 4	AdaDelta + MAE
Selection 5	Momentum + MSE
Selection 6	Momentum + MAE
Selection 7	SDG + MSE
Selection 8	SDG + MAE

**Figure:**
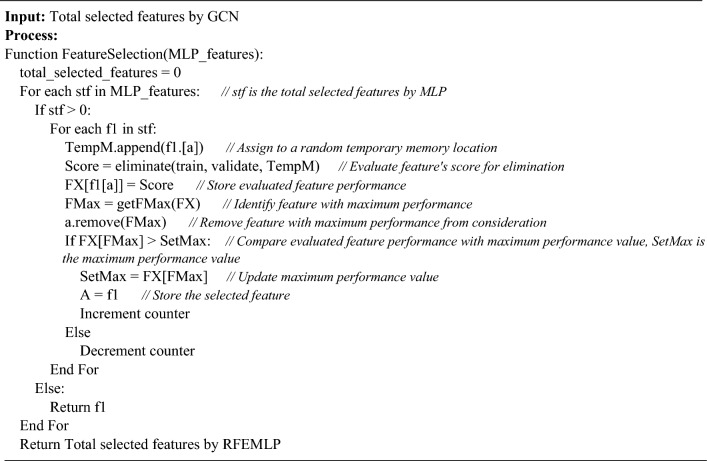
Algorithm 1 Feature selection using RFEMLP

### Sequence-based feature extraction

#### CodeBERT

The pre-trained models are effective in vulnerability prediction^108,109^. The CodeBERT combines bidirectional encoder representation from transformers and optimized BERT called RoBERTa^110^. The BERT is a self-supervised model that utilizes the characteristics of mask-based goals and a transformer-based architecture. The CodeBERT is the only large bimodal pre-trained model using natural and programming languages^111^. It effectively analyzes the semantic connections between programming language and eliminates the long-range dependency in code. Moreover, the multi-head attention mechanism of transformers effectively analyzes multiple key variables of data flow.

**Figure 6:**
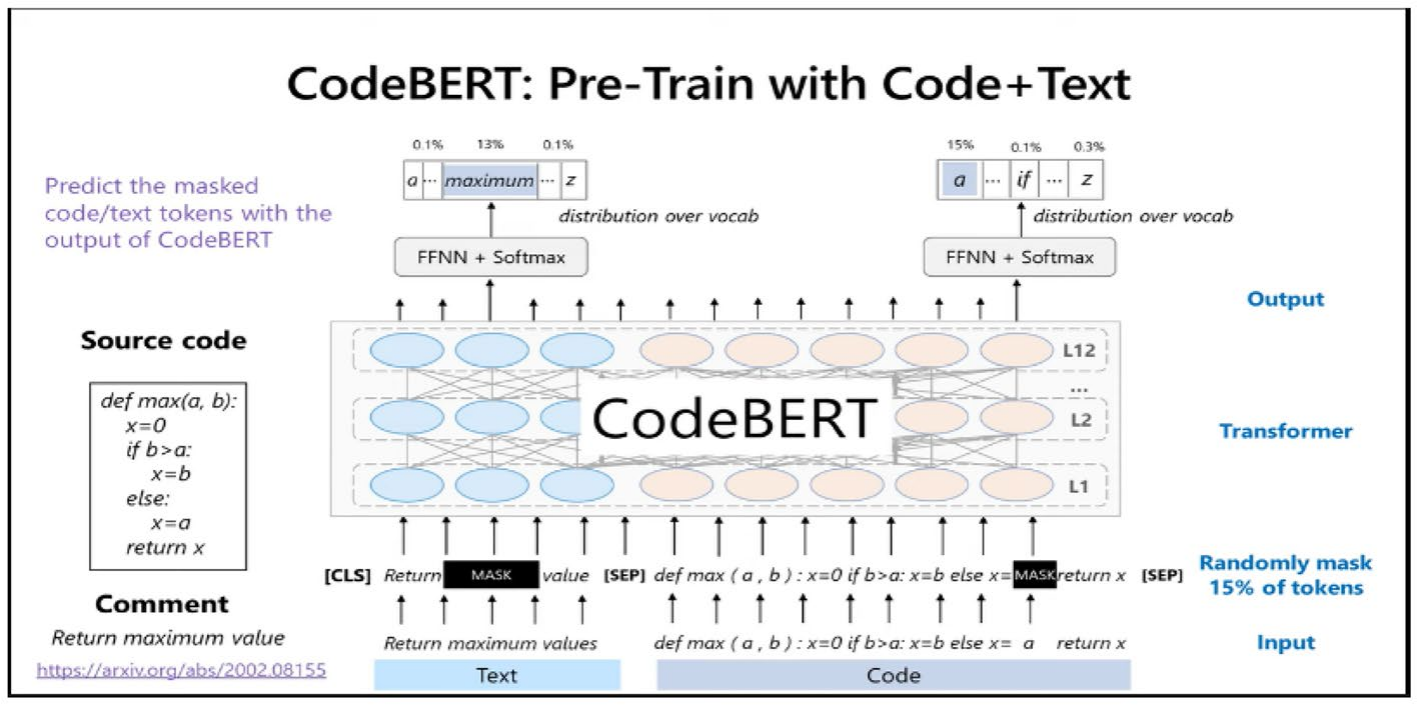
Structure of CodeBERT model.

The Fig. 6 illustrates the architecture of the CodeBERT model. In the first step, the CodeBERT takes code input and tokenizes the code. We have implemented the greedy longest match first algorithm for tokenizing. In the second step, the tokens are used to extract the features. To perform feature extraction, we have fine-tuned the CodeBERT by setting the batch size to 32, the learning rate of 10^–3^, 50 epoch size, and used early stopping to avoid overfitting.

### Classification

#### Quantum convolutional neural network with self-attentive pooling

The software Java source code has a complex lexical structure, and intricate syntactic and semantic features with longer length which is difficult to tackle. Moreover, the large and complex software can create computational and memory bottleneck issues while dealing with vulnerability detection. We have, therefore, employed a quantum neural network to overcome these issues with quantum mechanisms. The quantum mechanism is based on quantum entanglement and quantum superposition states. Quantum neural networks are embedding entanglement and quantum superposition states to improve the accuracy of neural networks. It utilizes the quantum bit, interference, superposion, and entanglement mechanism for information processing. The q-bit is a state vector depicted in the equation below

2 $$| \Psi \rangle = \theta | 0 \rangle + \delta |1 \rangle$$

where θ and δ are the probability amplitudes that are represented by complex numbers and |θ^2^| +|δ^2^|= 1. The quantum mechanism implies that any unitary matrix is a quantum gate U given below in Eq. (3).

3 $${\text{UU}}\dag \, = {\text{ U}}\dag {\text{U }} = {\text{ I}}$$

where U† is the conjugate transpose of a matrix U, and I is an identity matrix. There are three qubit gates 1. one qubit gate, which is a square root of NOT gate, also known as Pauli gates 2. two qubit gate which work on 4 × 4 unitary matrices; and 3. multiple-qubit gates which work on multiple qubits as 2n × 2n unitary matrices. The quantum mechanism resolved memory issues in huge computations and structural bottleneck issues and attained higher computing capabilities than classical computing.

The quantum convolutional neural network provides a promising machine learning paradigm. We have used a quantum pennyLane device to mimic the four-qubit device. The RY gate is responsible for converting the code into quantum bits. The quantum convolutional layer works as the conventional convolutional layer in the CNN model using a quantum computing mechanism. Quantum convolution works as small random quantum circuits (RQCs) to calculate convolution operation. It consists of three phases: encoding, RQC, and decoding. The RQC is applied to the convolutional layer and pooling layer. The encoding layer is responsible for converting the extracted features in classical form into a high-dimensional quantum bit state. We have applied basis encoding to convert the data into qubits. The concatenated features are converted into binary features and then into a quantum state. The embedded quantum state is the bit-wise conversion of binary string into a quantum subsystem; thus, the source code is transformed into the quantum bit. The paddle library in Python is used for basis encoding.

In the second layer, RQC is applied at a convolutional layer that uses multiple qubit gates among the adjacent qubit. Similarly, the qubit gates applied on pooling reduce the size of the quantum system. We have applied a self-attention mechanism on the pooling layer to improve the system's performance. The fully connected circuit is responsible for decoding and classifying the vulnerable code and the type of vulnerability identified. The QCNN uses multiscale entanglement MERA in the reverse direction and repeats until sufficiently reduces the size of the quantum system.

We have applied a novel pooling technique using a multi-head self-attention mechanism to improve the computation and memory footprints, thus improving the model's performance. The proposed self-attention mechanism comprised tokenization, multihead self-attention, spatial channel restoration, and sigmoid and soft max activation functions applied on the pooling layer to make it self-attentive. The input features are tokenized, and multi-head self-attention manages the long-term dependencies in the tokens, while the spatial channel restoration helps in decoding and restoring the tokens to self-attention maps. The activation function softmax rectifies the self-attention maps. Adding a self-attention mechanism in QCNN further improves the memory footprints and computation. The quantum convolutional neural network classifies the vulnerable code and identifies the vulnerability type.

**Figure 7:**
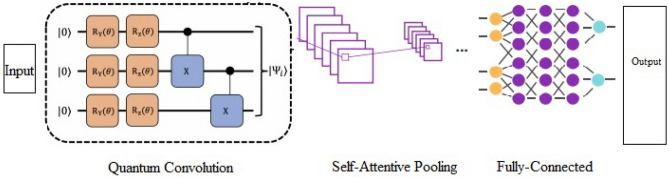
Structural composition of quantum neural network with self-attentive pooling.

The Fig. 7 above illustrates the overall structure of self-attentive QCNN model proposed to identify the security vulnerability and type of vulnerability.

**Figure:**
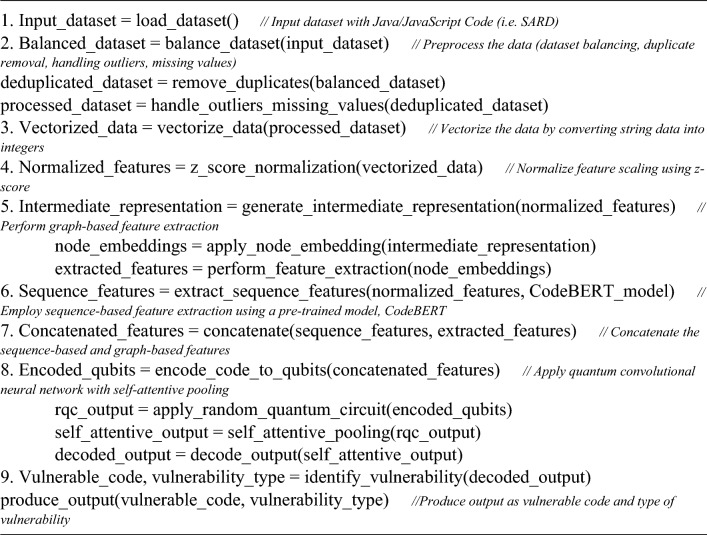
Algorithm 2 Composition of the proposed vulnerability detection system

## Experiments and results

### Experimental setup

The proposed automatic vulnerability detection system was evaluated via numerous experiments on a Windows-based computer equipped with an Intel^®^ Core™ i7-10700H processor and 128 GB of RAM. The model is implemented using Python and Tensorflow framework using library packages like Keras, NumPy, sci-kit-learn, and Pandas. The hyper-parameters are set as epoch = 50, learning rate = 0.005, momentum = 0.9, dropout rate = 0.3, loss = cross-entropy.

### Performance metrics

We assessed the performance of the proposed system using various metrics, including recall, precision, and accuracy. Accuracy was calculated according to Eq. (4).

4 $$\text{Accuracy} = \frac{\text{TP + TN}}{\text{TP + TN + FP + FN}}$$

In this equation, TN stands for true negative, TP for true positive, FP for false positive, and FN for false negative. Additional metrics employed for performance validation were precision (see Eq. 5), which represents the fraction of correct positive predictions, and recall (see Eq. 6), which indicates the ratio of correct positive predictions with all positive predictions.

5 $$\text{Precision} = \frac{\text{TP}}{\text{TP + FP}}$$

6 $$\text{Recall} = \frac{\text{TP}}{\text{TP + FN}}$$

### Comparative analysis

The proposed system is developed to effectively predict the software systems' security vulnerability. To analyze the performance of the proposed system, it underwent testing on source code to identify potential security vulnerabilities.

The Table 3 compares our technique with other deep learning techniques like CNN, SVM, GNN, LSTM, BiLSTM, ANN, MLP, DNN, and FFDNN. The proposed model displayed superior accuracy, precision, and recall, suggesting its enhanced effectiveness in detecting maximum security vulnerability.

**Table 3:** Comparative analysis with machine learning techniques.

Classifier	Accuracy	F1
CNN^112^	0.92	0.92
SVM^113^	0.96	0.95
GNN^114^	0.95	–
LSTM^115^	0.96	0.96
BiLSTM^116^	0.96	–
ANN^117^	0.98	0.98
MLP^118^	0.84	–
DNN^119^	0.82	–
FFDNN^120^	0.77	–
Proposed model	0.99	0.97

The research focused on different types of vulnerability, each possessing unique semantic features. The proposed system underwent training with the balanced SARD dataset containing synthesized data, making it universally applicable to various vulnerability types. To effectively assess the validity and performance of our system, the system was trained using other datasets, including Juliet Java 1.3, FUNDED, Vul4J, and CVEfixes. The SARD and Juliet java 1.3 are benchmark datasets made public by NIST.

The Table 4 depicts that the proposed system performed well with the other datasets FUNDED, Vul4j, CVEfixes, CodeXGLUE, SARD, VUDDY, and Julia jave 1.3, which proves the proposed system's validity.

**Table 4:** Performance evaluation of the proposed vulnerability detector using well-known datasets.

Dataset	Precision	Recall	F1 score
FUNDED	0.95	0.92	0.91
Vul4J	0.96	0.96	0.95
CVEfixes	0.98	0.95	0.93
CodeXGLUE	0.96	0.98	0.99
SARD	0.98	0.97	0.95
VUDDY	0.92	0.97	0.97
Juliet java 1.3	0.98	0.95	0.96

In Table 5 our proposed model is compared with the commercial vulnerability detection tools VulDeepecker, SQVDT, Exp-Gen, PreNNsem, ISVSF, VULDEFF, SedSVD, VulANalyZeR, FUNDED, GraphSPD, BiTCN_DRSN, and VERI. The proposed system outperformed in accuracy, precision, and recall rates.

**Table 5:** Comparative analysis with existing vulnerability detector.

	Accuracy	F1 Score	Precision	Recall
VulDeepecker^121^	0.95	0.93	0.92	0.94
SQVDT^122^	0.97	0.95	0.94	0.96
Exp-Gen^123^	–	–	0.95	–
PreNNsem^124^	0.96	0.97	0.96	0.98
ISVSF^125^	0.95	0.90	–	–
MFXSS^126^	0.98	0.98	0.97	0.96
VULDEFF^127^	–	0.88	0.91	0.85
SedSVD^128^	0.91	0.95	–	–
VulANalyZeR^129^	0.89	0.90	0.85	0.95
FUNDED^130^	0.92	–	–	0.94
GraphSPD^131^	0.80	–	–	–
BiTCN_DRSN^132^	0.95	0.95	0.92	0.98
VERI^133^	0.92	0.93	0.94	0.91
Proposed model	0.99	0.97	0.98	0.96

**Figure 8:**
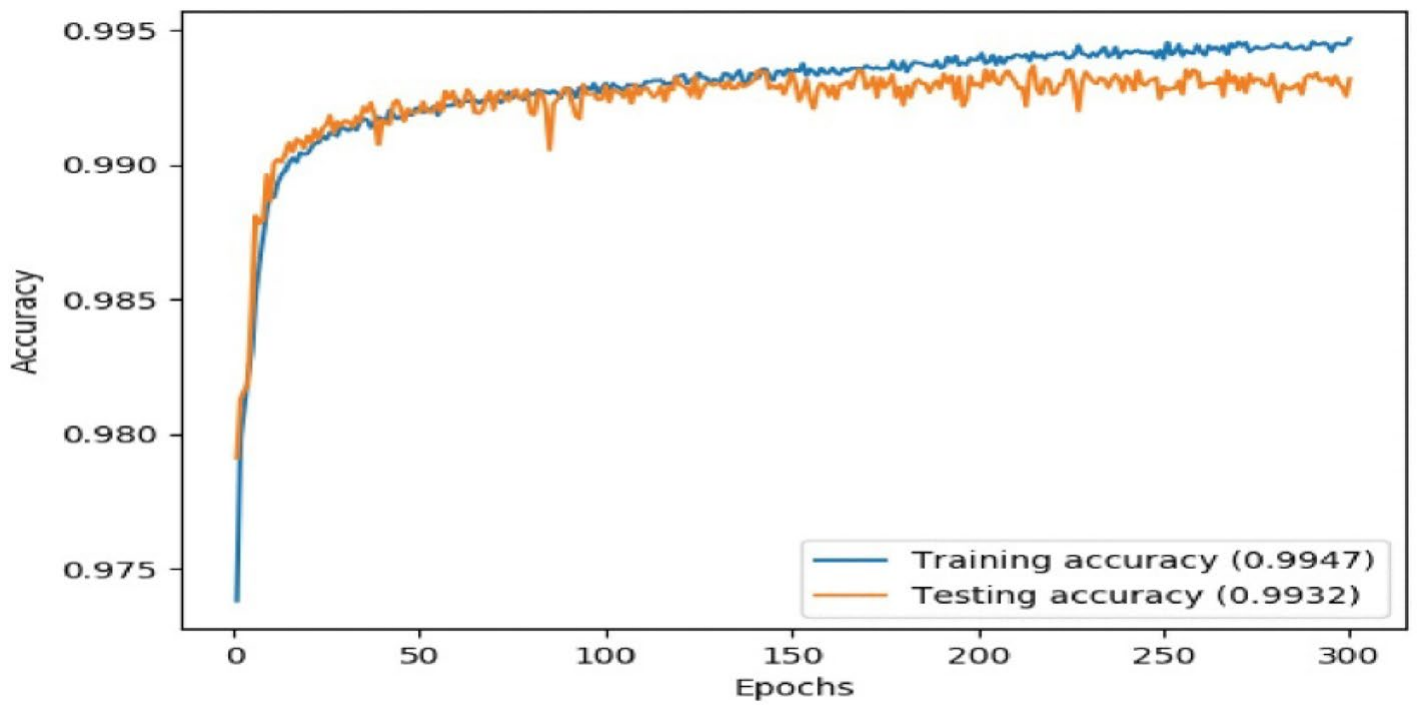
The training and test accuracy of the proposed system.

The Fig. 8 shows the proposed system's training and test accuracy. Data underscores the superior performance of our system, achieved by integrating hybrid feature extraction with syntax and semantic information of the code. Notably, our system successfully reduced the false-positive rate while ensuring a minimum number of missing values.

## Conclusion

This study proposes an innovative system designed to analyze vulnerability in software code, aiming to address limitations found in previous deep learning techniques. The vulnerability detection methods have fallen short in considering code semantics, leading to suboptimal performance. Our proposed system, combining graph-based feature extraction and sequence-based feature extraction with a proposed novel GCN-RFEMLP neural network, pre-trained model CodeBERT, and QCNN-self-attentive pooling, successfully audits source code for any potential security vulnerabilities. We leverage intermediate code representation, using a code property graph (CPG) for graphical code representation, consisting of an abstract syntax tree (AST), control flow graph (CFG), and program dependency graph.

The dataset is preprocessed considering the importance of data balancing, duplicate code removal, missing values, handling outliers, vectorization, and normalization for robustness, efficiency, and computational speed. Moreover, a quantum convolutional neural network with self-attentive pooling is used as a classifier. Our research concentrates on specific types of vulnerability: improper input validation, cross-site scripting (XSS), missing authorization, integer overflow, and SQL injection, which are listed among the top 25 most significant software security vulnerabilities in the common weakness enumeration (CWE). The Software Assurance Reference Dataset (SARD), a benchmark dataset, was employed to train our model. Furthermore, to prove the system's validity, the proposed system is used with other benchmark datasets, including FUNDED, Vul4j, CVEfixes, CodeXGLUE, SARD, VUDDY, and Juliet Java 1.3.

To validate the efficiency of our system, we compared its performance against not only prevalent deep learning approaches like CNN, SVM, GNN, LSTM, BiLSTM, ANN, MLP, DNN and FFDNN but also other available systems such as VulDeepecker, SQVDT, Exp-Gen, PreNNsem, ISVSF, VULDEFF, SedSVD, VulANalyZeR, FUNDED, GraphSPD, BiTCN_DRSN, and VERI. The results from our experiments demonstrate the superior performance of our proposed system across various metrics, signifying a promising advancement in the field of automatic vulnerability detection.

## Future directions

The proposed security vulnerability detection system, with its efficient feature extraction and quantum mechanism, including self-attentive pooling, successfully addresses existing issues in vulnerability detection in Java source code. While the system is tailored for the structural complexities of Java source code, extending the proposed mechanism to other programming languages is a crucial future direction to assess its effectiveness across diverse codebases. Additionally, exploring the applicability of the proposed system in resolving natural language processing (NLP) tasks holds promise for mitigating time, cost, and memory bottleneck issues in broader contexts.

## Data Availability

The datasets generated and/or analysed during the current study are available in the Github repository using the link https://github.com/Vul-Detect-Code/Vul-Detect.
